# A Card

**Published:** 1868-04-15

**Authors:** 


### *A Card.*

We smell a little brimstone and saltpetre under the following, which comes to us from Nashville:

A CARD.—As Rev. J. B. Lindsley and Doctors C. K. Winston, W. T. Briggs and W. K. Bowling have attempted to divest me, in an illegal manner, of my rights as a Professor in the Medical Department of the University of Nashville, and have made a parade of the matter in the newspapers, I hereby inform the public that I will seek redress in due time, through the courts of justice.

T. R. JENNINGS, M.D.,
*Prof. Anat. in Med. Col. Univ. of Nashville.*

"In the name of the Prophet—Figs!" we command the peace. This is a vermilion edict.

